# Effects of Na_V_1.5 and Rac1 on the Epithelial-Mesenchymal Transition in Breast Cancer

**DOI:** 10.1007/s12013-024-01625-x

**Published:** 2024-12-14

**Authors:** Zhuocen Zha, Fei Ge, Na Li, Shijun Zhang, Chenxi Wang, Fuhong Gong, Jingge Miao, Wenlin Chen

**Affiliations:** 1 First-Class Discipline Team of Kunming Medical University, Third Department of Breast Surgery, Peking University Cancer Hospital Yunnan, Yunnan Cancer Hospital, The Third Affiliated Hospital of Kunming Medical University, Kunming, Yunnan 650118 China; 2 Oncology department, Guizhou Hospital of the First Affiliated Hospital, Sun Yat-sen University, Guiyang, Guizhou 550000 China; 3 Department of Breast Surgery, The First Affiliated Hospital of Kunming Medical University, Kunming, Yunnan 650032 China

**Keywords:** Breast cancer, EMT, Na_V_1.5, Rac1

## Abstract

Breast cancer is a disease that seriously endangers the health of women. However, it is difficult to treat due to the emergence of metastasis and drug resistance. Exploring the metastasis mechanism of breast cancer is helpful to aim for the appropriate target. The epithelial-mesenchymal transition (EMT) is an important mechanism of breast cancer metastasis. Sodium channel 1.5(Na_V_1.5) and the GTPase Rac1 are factors related to the degree of malignancy of breast tumors. The expression of Na_V_1.5 and the activation of Rac1 are both involved in EMT. In addition, Na_V_1.5 can change the plasma membrane potential (Vm) by promoting the inflow of Na^+^ to depolarize the cell membrane, induce the activation of Rac1 and produce a cascade of reactions that lead to EMT in breast cancer cells; this sequence of events further induces the movement, migration and invasion of tumor cells and affects the prognosis of breast cancer patients. In this paper, the roles of Na_V_1.5 and Rac1 in EMT-mediated breast cancer progression were reviewed.

## Introduction

Currently, breast cancer(BC) has become the world’s second-largest cancer after lung cancer, with an estimated 2.3 million new cases each year [1]. There are many mechanisms involved in the proliferation, invasion and metastasis of breast cancer tumors, among which the epithelial-mesenchymal transition (EMT) plays an important role [2], and changes in the ion channels and their related transcription factors are very important for its regulation [3, 4]. The expression of the sodium channel Na_V_1.5 in highly invasive breast cancer cells is significantly higher than that in less invasive breast cancer cells. Na_V_1.5 has been found to be positively correlated with the invasiveness of breast cancer, and high expression of Na_V_1.5 can promote cell membrane depolarization and further activate the guanosine triphosphatase (GTPase) Rac1, which is one of the mechanisms that promotes EMT [5]. This paper reviews the role of Na_V_1.5 and Rac1 in EMT-mediated breast cancer progression.

## The Mechanism of EMT in the Development of Breast Cancer

EMT refers to the process by which epithelial cells with polarity are transformed into mesenchymal cells with the ability migrate [6]. EMT involves the loss of cell polarity, the reduction of intercellular contacts, cytoskeleton restructuring and protein degradation in the extracellular matrix [7–10]. Because of the disappearance of the interaction between epithelial cells, the intercellular structure between these tissues becomes loose; at the same time, strong abilities to move, infiltrate into local tissues, invade blood vessels and lymphatic vessels, and distant metastases are gained [10, 11]. When metastatic cancer cells colonize, mesenchymal epithelial transformation (MET) can occur to achieve redifferentiation [12, 13]. Notably, EMT involves many signaling pathways and regulatory molecules, such as TGFβ, Wnt/β-catenin, Hedgehog, and Notch [14–16]. Recent studies have reported that inflammatory cytokines can activate EMT [17].

During EMT, the epithelial marker E-cadherin is downregulated or lost, mesenchymal markers such as N-cadherin, vimentin and fibronectin are upregulated, and matrix metalloproteinases (MMPs) are secreted [18, 19].The absence of E-cadherin is particularly important in this process. E-cadherin can be used as both an intercellular adhesion molecule and a typical negative regulator of the Wnt signaling cascade in epithelial cells E-cadherin is mainly regulated by epithelial mesenchymal transition factor (EMT-TF), which includes the basic helix-loop-helix (BHLH) family members twist and the E2A gene products E12/47,SNAIL family members Snail1 and Snail2, and zinc finger homeobox (ZFH) family members ZEB1 and ZEB2 [20–22]. EMT-TFs are often triggered by inflammatory, physical constraints, metabolic stress, abnormal activation of signal pathways, etc [23]. This promotes malignant transformations and the development of primary tumors in cooperation with mitogen oncoprotein, which also promotes malignant transformation by inhibiting the p53 pathway and providing cancer cells with stem cell characteristics and cell plasticity [24].

Mediated by calcium ions (Ca^+^), E-cadherin adheres to homologous molecules to connect adjacent epithelium and plays a role through γ-cadherin, β-cadherin and P120-cadherin [25]. F-actin is the key to coordinating cell motility and E-cadherin functions by linking to it in an indirect manner. That is, E-cadherin is linked to β-catenin which is able to bind α-catenin, which can bind F-actin [26]. RhoA/Rac1/Cdc42 is the key molecule in cytoskeletal regulation. β-catenin can cross-react with Rac1. P120-catenin regulates the GTPase RhoA/Rac1/Cdc42 while being able to bind to E-cadherin and promote aggregation [27]. E-Cadherin transformation causes epithelial cells to lose their adhesion between cells and gain affinity for interstitial cells, which improves cell mobility and invasion. These changes occur in tumor cells and are beneficial for tumor cell invasion into blood vessels and adjacent tissues to promote the distant tumor metastasis [20].

EMT in the early stage of carcinogenesis is due to alterations in key gene expression patterns, which leads to a cascade of changes in cells, molecules and cell morphology [28, 29]. That is, there is large-scale remodeling of the cytoskeleton, including transformation from the main cytokeratin into the intermediate silk network rich in vimentin, microtubule deproteinization that promotes the formation of microantennae, and the increases in the expression of proteolytic enzymes and extracellular matrix components [30, 31]. Breast cancer cells with EMT show antiaging, apoptosis, and resistance to treatment [32–35]. EMT affects not only the tumor cells themselves but also the TME. In the early stage of transformation, the EMT process induces cancer cells to secrete a large number of matrix proteins, angiogenic factors and anti-inflammatory factors into the TME. This ultimately leads to connective tissue hyperplasia, tissue fibrosis, angiogenesis and immune escape, thus promoting tumor progression, invasion and metastasis [36, 37].

## The Role of Na_V_1.5 in EMT

**Fig. 1:**
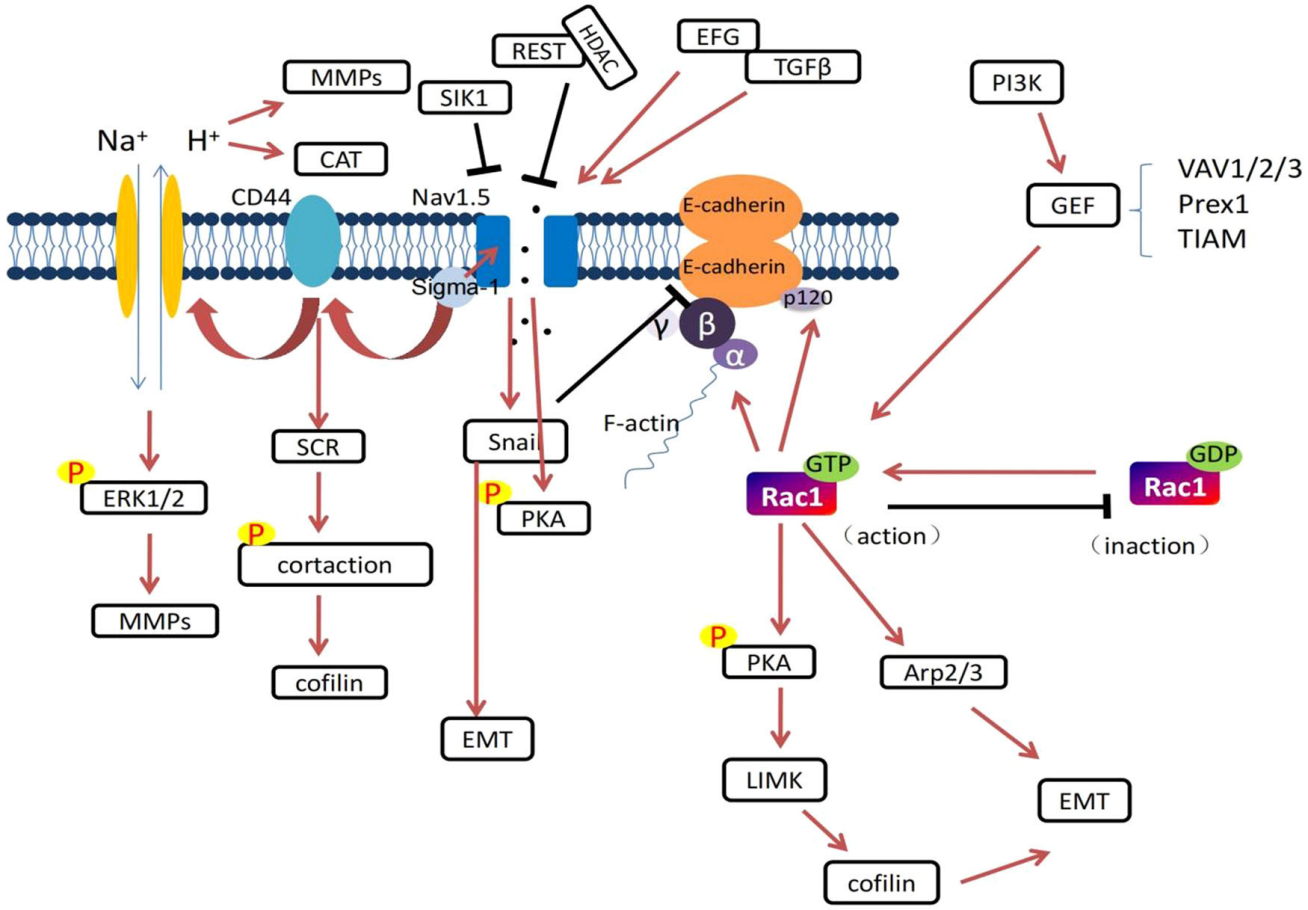
The red arrows in the figure all indicate promotion, while the“┤”indicates inhibition. (1) The Na_V_1.5 channel can induce Na^+^ inflow and the plasma membrane potential (Vm) depolarization and activate Rac1. Activated Rac1 can act on F-actin, promote epithelial-mesenchymal transition (EMT) and enhance the invasiveness of tumor cells. E-cadherin adheres to homologous molecules to connect adjacent epithelium and plays a role through β-cadherin, γ-cadherin and P120 cadherin. The final result is the connection of F-actin with α-cadherin to promote EMT. (2) Na_V_1.5 can be activated by EFG and TGFβ, sigma-1 can promote Na_V_1.5, HDAC is recruited by SIK1, REST can inhibit Na_V_1.5, and activated Nav1.5 can promote Snail, which plays a key role in EMT. In addition, Na_V_1.5 promotes CD44 and nhe-1 to participate in other biological effects in cells. (3) Rac1 in its active form binds GTP, while when combined with GDP, it is inactive, changing between the two states. Generally, Rac1 is mainly activated by GEF in the PI3K pathway in breast cancer, and GEF includes the molecules VAV1/2/3, Prex1, TIAM, etc. Activated Rac1 regulates PKA phosphorylation to make downstream LIMK and cofilin participate in EMT and also regulates Arp2/3 to participate in this process

Na_V_1.5 (encoded by the SCN5A gene) is one of the nine members of the Voltage-gated sodium channel (VGSC) family. In breast cancer, the Na_V_1.5 isoform, encoded by SCN5A, is predominantly expressed. Na_V_1.5 generates sodium currents in the cell membrane, thereby regulating cellular functions related to the invasive ability [38]. Overexpression of Na_V_1.5 allows continuous entry of Na^+^ into cells and promotes extracellular matrix degradation and cancer cell invasion through a series of downstream signaling pathways [39, 40]. Na^+^ influx mediated by Na_V_1.5 channels increases Na^+^/H^+^ exchanger-1 (NHE-1) activity, thereby promoting H^+^ efflux and increasing Na+ entry into cancer cells, leading to intracellular pH alkalinization and extracellular pH acidification [40–42]. Acidification of the pericellular microenvironment favors the enhanced activity of cysteine cathepsin (Cat) and matrix metalloproteinase (MMP), which digest the extracellular matrix and increase the invasiveness of breast cancer cells [40–43]. (Fig. 1) Na_V_1.5 regulates the aggressiveness of breast cancer cells through multiple mechanisms. Na_V_1.5 can enhance the invasion of breast cancer cells through the CD44-src-cortactin signaling axis. Upregulated expression of Na_V_1.5 can increase the level of the CD44 protein. Once CD44 adheres to its ligand, it can activate SRC and control the phosphorylation of cortical actin [44]. The release of cofilin, which leads to actin breakage, results in cells being able to extend into the extracellular matrix and play proteolytic role [42, 45]. In addition, CD44 upregulates the expression and activity of NHE-1, leading to increased expression levels of MMPs 2, 9, and 14, which facilitates extracellular matrix degradation The Na^+^ current carried by Na_V_1.5 can also phosphorylate protein kinase A(PKA) so that the level of Na_V_1.5 mRNA increases. As a result, the level of Na_V_1.5 on the cell membrane increases and that in the cytoplasm decreases. Externalization of Na_V_1.5 enhances the invasion and migration of breast cancer cells, while an increase in Na_V_1.5 on the membrane enhances the Na^+^ current and forms a positive feedback loop [46]. Snail is the key transcription inhibitor of E-cadherin in breast cancer EMT. Snail recruits specific chromatin-modifying and chromatin-remodeling complexes to the E-cadherin promoter to silence the expression of E-cadherin and induce EMT during tumor progression [47]. After knocking out SCN5A in MDA-MB-231 cells, the expression of SNAI1 in the cells was significantly reduced, the filamentous foot length was shortened, the number was reduced, and the invasiveness was weakened [48], and the expression level of SNAI1 in breast cancer cells overexpressing Na_V_1.5 increases. Na_V_1.5 can also depolarize the plasma membrane (Vm), regulate Rac1 and promote EMT [49] (Fig. 1).

Voltage-gated sodium (Na^+^) channels form heteromeric proteins consisting of α subunits formed by macropores and small auxiliary β subunits. Na_V_1.5 is encoded by the SCN5A gene, which encodes the α subunit. According to research reports, SCN5A mutations can cause a variety of Pathophysiology phenotypes, such as long QT Type 3(LQT3), Brugada syndrome syndrome (BRS) and heart conduction disease (CCD). Recent data has provided compelling evidence for the involvement of multiple voltage-gated Na+ channels in the metastatic behavior of various types of cancer cells, including breast cancer [39, 50]. Specifically, in breast cancer, a splice variant known as “neonatal” Na_V_1.5 or nNa_V_1.5, has been identified as a key player in enhancing metastatic cell behavior [50]. This variant is notably up-regulated in MDA-MB-231 cells, a highly metastatic human breast cancer cell line, and gives rise to a TTX-resistant cardiac-type Na^+^ current [51]. Furthermore, elevated levels of nNa_V_1.5 have been observed in human breast cancer biopsy tissues, with a significant correlation found between Na_V_1.5e expression and lymph node metastasis [50]. Experimental interventions, such as treatment with 10 µM TTX and specific down-regulation of Na_V_1.5e in MDA-MB-231 cells using siRNA or a specific antibody, have demonstrated the essential role of this splice variant in promoting migration and invasion. In contrast, the Na^+^ channel β1 subunit is prominently expressed in MCF-7, where its function as a cell adhesion molecule appears to impede tumor cell migration. Interestingly, down-regulation of β1 in MCF-7 cells led to an increase in nNa_V_1.5 expression, resulting in reduced cell adhesion and enhanced migration, effects that were sensitive to TTX [50, 51]. While the exact relationship between Na_V_1.5e expression and metastasis remains unclear, both Na_V_1.5e and β1 hold promise as early indicators of metastasis and potential therapeutic targets for combating metastatic cancers.

Na_V_1.5 is regulated by many factors. The repression element silence transcription factor (REST) recruits histone deacetylase (HDAC) to inhibit transcription. Downregulation of HDAC2 expression can enhance Na_V_1.5 expression, thus promoting tumor invasiveness [52]. Salt-inducible kinase 1 (SIK1) is also a repressor, and its reduced expression promotes Na_V_1.5-dependent invasiveness and expression of the EMT-associated transcription factor SNAI1 [53]. The sigma-1 receptor binds to the Na_V_1.5 protein in breast cancer to form a compound with quadruple symmetry, which shifts the Na_V_1.5 protein to the plasma membrane, thus increasing metastatic activity [54, 55]. The expression of salt-induced kinase 1(SIK1) is low in different breast tissues, and a decrease in SIK1 expression can promote invasiveness that is dependent on Na_V_1.5 and the expression of the EMT-related transcription factor SNAI1 [53]. Epidermal growth factor (EGF) can enhance the expression of Na_V_1.5 channels to improve the migration ability of cells [56]. The expression of the estrogen receptor-α (ERA) also has an effect on Na_V_1.5, showing an inhibitory effect [57, 58]. There are many studies on Na_V_1.5 inhibitors (Table 1), but most of them are for the treatment of neurological and cardiovascular diseases. With the deepening of research on the mechanism of Na_V_1.5 in cancer, Na_V_1.5 inhibitors have great application prospects in the treatment of cancer, especially breast cancer.

**Table 1:** Inhibitors of Na_V_1.5

Product name	Type	Biological activity	Research area	Reference
Lidocaine	compound	blocking Na_V_1.5-mediated EMT and FAK/Paxillin signaling pathway	Ovarian Cancer	[113]
Eicosapentaenoic acid	organic compound	blocking the Na_V_1.5 sodium channel	Ovarian Cancer	[118]
Phenytoin or tetrodotoxin	organic compound	targeting Na_V_1.5 sodium channels	Breast Cancer	[85, 119]
FS50 protein	protein	decreasing Na_V_1.5 mRNA expression	Breast Cancer	[120]
n-3 polyunsaturated docosahexaenoic acid	organic compound	inhibiting Na_V_1.5 and NHE-1 pro-invasive activities	Breast Cancer	[121]
Propranolol	organic compound	inhibiting neonatal Na_V_1.5 activity	Breast Cancer	[122]
Docosahexaenoic acid	organic compound	blocking voltage-gated sodium channel activity	Breast Cancer	[123]
Taxol	organic compound	exerting anti-tumoral activities, in cells expressing Na_V_, at low doses	Breast Cancer	[124]
Flecainide acetate (R-818)	compound	blocking the Na_V_1.5 sodium channel	Cardiovascular Disease	[125]
Flecainide-d4 acetate(R-818-d_4_)	compound	blocking the Na_V_1.5 sodium channel	Cardiovascular Disease	[126, 127]
Jingzhaotoxin-III (β-TRTX-Cj1α)	polypeptide	selective blocker of Na_V_1.5 channels	Neurological Disease	[128]
Jingzhaotoxin XI (JZTX-XI)	polypeptide	selective blocker of Na_V_1.5 channels	Neurological Disease	[129]
Phlo1a (μ-TrTx-Phlo1a)	polypeptide	selective Na_V_1.7 inhibitor, has a weak inhibitory effect on Na_V_1.2 and Na_V_1.5	Neurological Disease	[130]
Phlo1b (μ-TrTx-Phlo1b)	polypeptide	selective Nav1.7 inhibitor, has a weak inhibitory effect on Na_V_1.2 and Na_V_1.5	Neurological Disease	[130]
VGSC blocker-1	compound	small molecule blocker of neonatal isoform of Na_V_1.5	Cancer	[131]
Lu AE98134	compound	an activator of voltage-gated sodium channels	Neurological Disease	[132]
Phrixotoxin 3	polypeptide	blocker of voltage-gated sodium channels	Neurological Disease	[133]
Phrixotoxin 3 TFA	polypeptide	blocker of voltage-gated sodium channels	Neurological Disease	[133]
GFB-8438	compound	selective TRPC5 inhibitor	Neurological Disease	[134]
Nav1.1 activator 1 (compound 4)	compound	Nav1.1 activator with BBB penetration, significant selectivity against Na_V_1.2, Na_V_1.5, and Na_V_1.6	Neurological Disease	[135]
GNE-0439	compound	Na_V_1.7-selective inhibitor, inhibits Na_V_1.5 with an IC_50_ of 38.3 μM.	Neurological Disease	[136]
ICA-121431	compound	voltage-gated sodium channel (Na_V_) blocker	Cardiovascular Disease	[137]
Relutrigine (PRAX-562)	compound	orally active inhibitor of persistent sodium channel.	Neurological Disease	[138]
Ceratotoxin-1 (CcoTx1)	polypeptide	voltage-gated sodium channel subtypes inhibitor	Neurological Disease	[133]

## The Role of Rac1 in EMT

The Rho GTPase is a small G protein that regulates the cytoskeleton, cell cycle processes and gene expression. The Rho GTPase superfamily contained 23 members, including Rac1,RhoA and Cdc42, which are the key factors involved in cell shape change. Rac1 promotes the formation of lamellar pseudopodia and membrane folds. RhoA participates in the formation of tension fibers and the assembly of adhesive spot complexes. Cdc42 promotes the formation of filamentous pseudopodia. The activities of these Rho GTPases can be inhibited by P120-catenin to maintain stable adhesion between epithelial cells [59]. Rac1, a RAS-related C3 botulinum toxin substrate, is the most characteristic Rho GTPase, and it switches between being in an active GTP binding conformation and an inactive GDP binding conformation; GTP is hydrolyzed into GDP through its inherent GTPase activity, thus inactivating the G protein [60]. In general, Rac1 can interact with the waveform regulation complex, promote actin nucleation of the branched actin filament network, and play an important role in the formation of platelet liposomes and cell migration.

Rac1 is activated by receptor tyrosine kinases (such as EGFR and HER2) or nonreceptor tyrosine kinases (such as SRC and FAK), G protein-coupled receptors (GPCRs) and integrins (such as α/β). The mechanism of Rac1 signaling in cancer is usually the result of an increase in upstream inputs from tyrosine kinase receptors (PI3Ks) or GEFs (such as VAV, Prex and TIAM) or a decrease inRac1 inactivation caused by GAP. In breast cancer cells,Rac1 is the downstream effector of the ERBB receptor, which mediates the migration reaction of ErbB1/EGFR ligands (such as EGF or transforming growth factor α) and ERBB3 ligands through GRB7-VAV2 [61].Rac1-GEF-Prex1 is an important mediator of the Rac1 reaction in breast cancer cells. Prex1 is highly overexpressed and amplified in human ductal breast tumors, especially those expressing ERBB2 and the estrogen receptor (ER). Prex1is activated by thePI3K products PIP3 and subunit Gβγ and integrates signals between theERBB2 receptor and GPCRs [62, 63]. The tumor cell phenotype mediated by the Wnt-β-catenin pathway of Rac1 GTPase signal transduction was found in triple-negative breast cancer [64]. After Rac1 is activated, it mainly participates in EMT of breast cancer through PAK. Active Cdc42 or Rac1 binds to the GBD domains of PAK (PAKs 1, 2 and 3) to enhance kinase activity [65]. Rac1-PAK1 signaling regulates rearrangement of the actin cytoskeleton and remodeling of the plasma membrane in response to extracellular stimuli by phosphorylating different proteins, including LIM kinase (LIMK)/Co-Filin, GP91 Phox/p67 Phox and AKT/bcl2/bcl-xL [66, 67]. Rac1 is also a potential upstream regulator of the Arp2/3 complex, which is necessary for the extension of lamellar liposomes and mediates cell migration [68]. Transforming growth factor-β (TGF-β) not only enhances cell motility through activation of RAC1 by Phosphatidylinositol 3-kinases, but its role is to synergize two independent pathways downstream of TGF-β type I receptors, thus enabling enhanced RAC1 activation signaling, resulting in increased cell movement [69]. (Fig. 1).

Dysregulation of normal Rac signaling is implicated in the pathogenesis of various diseases, with cancer being a notable example where hyperactivation of the Rac pathway contributes to increased cell migration and invasiveness. This dysregulation can manifest through different mechanisms, including up-regulation of Rac itself, expression of the active spliced variant Rac1b, or, albeit rarely, mutations in RAC genes [70, 71]. During the sequencing of Rac1 in breast tissues, a novel Rac1 isoform containing an insertion of 19 codons within the Rac1 reading frame proximal to switch region II was discovered and designated as Rac1b. Functionally, the Rac1b protein exhibits characteristics of a fast-cycling GTPase based on GTP binding and hydrolysis assays [72]. Rac1b is characterized by an additional 19-amino acid insertion located immediately following the switch II domain, a critical region for Rac1’s interactions with regulators and effectors. Recent investigations have revealed that Rac1b displays the biochemical traits of a constitutively active GTPase; however, it exhibits reduced affinity for downstream effectors, implying potential functional deficiencies. Notably, Rac1b demonstrates heightened intrinsic guanine nucleotide exchange activity, compromised intrinsic GTPase activity, and an inability to interact with RhoGDI. Functionally, Rac1b has been shown to drive growth transformation in NIH3T3 cells and disrupt density-dependent and anchorage-dependent growth. Moreover, Rac1b facilitates the activation of the AKT serine/threonine kinase. This suggests that Rac1b may selectively engage a specific subset of Rac1 downstream signaling pathways to modulate cellular processes [73].

The activation of Rac1 is a key step in the EMT mechanism [74]. The rigidity of the tumor matrix controls downstream signals by inducing Rac1 and Rac1b, which play an important role in promoting EMT. Flexible matrix and normal breast tissue can protect against EMT, while a rigid matrix can promote EMT-induced breast tumors. The rigid matrix is favorable for Rac1b to localize to the plasma membrane and form a complex with NADPH oxidase. This complex can promote the production of reactive oxygen species (ROS), the expression of SNAI1 and activation of the EMT program. In contrast, a flexible matrix inhibits membrane localization and the subsequent redox changes in Rac1b [75]. Rac1b can mediate EMT and genomic instability induced by MMP3 by producing ROS in certain microenvironments, thus increasing the malignant transformation of breast cancer cells [75].Under pathological conditions, Rac1 activation can mediate EMT in cells [76] and induce invasion and metastasis of a breast cancer cell line model in vivo by promoting cell migration [77]. In addition, Rac1 is associated with tumor angiogenesis [78], the promotion of cell survival under endoplasmic reticulum stress [79], regulation of the survival of glucose-independent cells [80], and tumor drug resistance [81, 82]. Rac1is upregulated in chemotherapy-resistant breast cancer and indicates that the prognosis and neoadjuvant chemotherapy results will bepoor. Rac1 gene knockout increases chemotherapy sensitivity and reduces chemotherapy resistance in breast cancer [83].

## Na_V_1.5 Activates Rac1 to Induce EMT

Na_V_1.5 and Rac1, although they do not interact directly, exhibit significant functional connections in cancer invasion and metastasis by regulating shared signaling pathways and biological processes such as ion flow, cytoskeletal reorganization, and EMT (epithelial-mesenchymal transition). The Vm is related to the cell cycle process and the differentiation, development, regeneration and migration of cancer cells. The Vm of proliferating tumor cells is depolarized, while the Vm of the terminal differentiated nontumor cells is hyperpolarized [84]. Na_V_1.5 can produce a continuous inward Na^+^ current, which depolarizes the Vm in resting cells [85]. Vm depolarization promotes the redistribution of anionic phospholipid PIP2 and phosphatidylserine in the plasma membrane; this leads to the activation of Rac1 and further promotes EMT and regulates cell morphology and migration in breast cancer cells [85]. In non-excitable cancer cells, Na_V_1.5 generates a persistent inward Na^+^ current, leading to membrane potential depolarization at a steady state [85]. The activity of Na_V_1.5 promotes cell motility and invasive behavior through various mechanisms, including the activation of MAPK signaling pathways [86]. Notably, the persistent inward Na^+^ current carried by Na_V_1.5 induces membrane potential depolarization over minutes, resulting in enhanced colocalization of Rac1 with phosphatidylserine at the leading edge of migrating cells, thereby facilitating Rac1 activation. Utilizing a genetically encoded FRET biosensor for Rac1 activation, it is observed that depolarization-induced Rac1 activation leads to the acquisition of a motile phenotype. Recent studies have also demonstrated that Na_V_1.5 is able to mediate VM depolarization, which in turn regulates RAC1 activation, allowing cells to migrate in response to ionic changes in EMT [87]. (Fig. 1).

The interaction between Na_V_1.5 and Rac1 also involves various small molecules and proteins, which play crucial roles in regulating cell migration, invasion, and other cancer-related processes. (1) Sodium-Hydrogen Exchanger 1 (NHE1): NHE1 plays an essential role in the interaction between Na_V_1.5 and Rac1. Activation of Na_V_1.5 leads to an increase in intracellular sodium ion concentration. NHE1 helps maintain intracellular pH balance by expelling sodium ions and absorbing hydrogen ions. The rise in intracellular pH enhances the activity of proteases, such as matrix metalloproteinases (MMPs), which contribute to the degradation of the extracellular matrix, thereby promoting cell motility. Na_V_1.5 activation leads to sodium influx, while NHE1 activation promotes Rac1 activity through pH regulation, further enhancing the cell’s invasiveness. NHE1 forms a linked pathway with Na_V_1.5 and Rac1, collectively driving cell migration behavior [87, 88]. (2) Calmodulin: Calmodulin is a calcium-dependent signaling molecule that is widely involved in regulating calcium-related signaling pathways. Rac1 activation is modulated by calcium signaling, with calmodulin playing a key role in this process. The sodium influx through Na_V_1.5 can indirectly regulate intracellular calcium concentration via the sodium-calcium exchange mechanism. Calmodulin senses changes in calcium concentration and interacts with Rac1, promoting Rac1 activation. Calcium signaling further regulates actin reorganization, thereby enhancing the cell’s migratory ability [89, 90]. (3) Matrix Metalloproteinases (MMPs): MMPs are a class of enzymes involved in the degradation of the extracellular matrix (ECM) and play a critical role in cancer metastasis. The synergistic action of Na_V_1.5 and Rac1 is often accompanied by the activation of MMPs, which promote the breakdown of the ECM, making it easier for cancer cells to invade surrounding tissues. The sodium influx mediated by Na_V_1.5 regulates intracellular pH through pathways like NHE1, leading to MMP activation. MMPs play a central role in the degradation of the ECM and the invasion process in cancer cells. Rac1, on the other hand, promotes cell migration through cytoskeletal reorganization, allowing the cells to move freely through the degraded matrix [91–93]. (4) Phosphoinositide 3-Kinase (PI3K)/Protein Kinase B (AKT) Pathway: The PI3K/AKT pathway is an important signaling pathway for cell survival, proliferation, and migration, and Rac1 can promote cell migration and EMT processes through this pathway. In some cases, the interaction between NaV1.5 and Rac1 can enhance cancer cell migration and invasion by activating the PI3K/AKT pathway. Once Rac1 is activated, it can directly interact with AKT kinase through PI3K, activating downstream signaling molecules that regulate cell survival and migration. Na_V_1.5, through its electrophysiological effects and ion channel regulation, provides a favorable electrochemical environment for this pathway [94–96]. (5) p21-Activated Kinase (PAK): PAK is one of the downstream effectors of Rac1, responsible for regulating the actin cytoskeleton and cell migration. Na_V_1.5 indirectly activates Rac1, which further promotes the activation of PAK, leading to cytoskeletal reorganization and cell movement. By modulating Rac1 activity, NaV1.5 indirectly activates PAK, and PAK, in turn, influences actin dynamics [97, 98]. (6) Actin-Binding Proteins (e.g., WAVE Complex): The WAVE complex is one of the key proteins involved in regulating actin polymerization. Once Rac1 is activated, it can directly interact with the WAVE complex to control the formation of lamellipodia and cell migration. Rac1 regulates actin reorganization through the WAVE complex, and Na_V_1.5 enhances the function of WAVE by influencing Rac1 activation levels, ultimately promoting cell morphological changes and migration [99, 100].

The interaction mechanism between Na_V_1.5 and Rac1 may also involve other signaling pathways that depend on Na_V_1.5 activity. For example, Na_V_1.5 can promote the activation of Src kinase and the phosphorylation of adhesion proteins and cofilin [42]. Therefore, Na_V_1.5-induced membrane potential (Vm) depolarization may activate Rac1, increase the phosphorylation of adhesion proteins, and thereby enhance cofilin activity and actin filament polymerization. Src has also been shown to regulate Rac1 activity, suggesting the possibility of feedback regulation [101]. Na_V_1.5 positively regulates the expression of the metastasis-promoting protein CD44, which may activate Src and thus promote this process [102–105]. Activated Rac1 regulates cytoskeletal remodeling by interacting with effector molecules such as PAK and WAVE, thereby promoting cell migration and invasion [42, 50, 105–107]. Rac1 itself may also regulate the function of Na_V_1.5 in return. Some studies suggest that Rac1 can influence the voltage-dependent activation of Na_V_1.5 through its downstream signaling molecules, such as the PI3K/AKT pathway, thereby regulating the activity of sodium channels. This feedback loop may further enhance the synergy between Na_V_1.5 and Rac1 [96, 108, 109].

The interaction between Na_V_1.5 and Rac1 constitutes a complex signaling network involving multiple key molecules, proteins, and shared upstream or downstream signaling pathways (such as PI3K/AKT, EGFR, and Ca^2+^-dependent pathways). Their synergy may be achieved through these common signaling pathways and promotes cancer progression by influencing mechanisms such as cytoskeletal remodeling, cell adhesion, extracellular matrix (ECM) degradation, and signal transduction [92, 110–112]. In promoting cancer progression, this interaction primarily operates through: (1) Epithelial-Mesenchymal Transition (EMT): The synergy between Na_V_1.5 and Rac1 can accelerate the EMT process. Na_V_1.5 indirectly activates Rac1 by regulating the ionic environment and potential changes inside and outside the cell, while Rac1 further drives cell migration, morphological changes, and the completion of the EMT process [87, 113–115]. (2) Promotion of Invasion and Migration: Na_V_1.5 indirectly activates Rac1 by promoting sodium influx and regulating calcium signaling, while Rac1 directly enhances the migration and invasion capabilities of cells through cytoskeletal remodeling. The synergy between the two is particularly pronounced in the process of increased invasiveness of cancer cells [87]. (3) Extracellular Matrix (ECM) Degradation: The degradation of the extracellular matrix is a critical step for cancer cells to breach tissue barriers and invade adjacent tissues. Na_V_1.5 and Rac1 play a role in this process by regulating the activity of matrix metalloproteinases (MMPs) [116, 117]. (4) Tumor Metastasis: Na_V_1.5 and Rac1 accelerate the metastasis of cancer cells by jointly regulating mechanisms of cell migration and invasion. Na_V_1.5 indirectly enhances Rac1 activity by modulating the electrophysiological environment and ion concentrations, while Rac1 directly regulates cell motility, ultimately promoting tumor metastasis [87, 88].

Although the specific roles of NaV1.5 and Rac1 in breast cancer progression have been partially elucidated, the metastasis and drug resistance of breast cancer involve the interactions of multiple signaling pathways and genes. Focusing solely on NaV1.5 and Rac1 may not fully encompass all mechanisms involved. Additionally, the expression and activity of NaV1.5 and Rac1 may be regulated differently at various stages of disease progression, meaning that studies at a single time point may not capture their dynamic changes throughout breast cancer progression. To overcome these limitations, more longitudinal studies and dynamic detection methods may be required. These limitations also highlight future research directions, including the need for broader clinical data support, more detailed analyses of different breast cancer subtypes, and the exploration of more comprehensive and personalized therapeutic strategies.

## Conclusion

Na_V_1.5 and Rac1 are both involved in EMT in breast cancer. Na_V_1.5 can also mediate the activation of Rac1, which indicates the feasibility of inhibiting Na_V_1.5 for the treatment of breast cancer. However, there is more than one activation mechanism for Rac1. Currently, the other activation mechanisms have not been compared, and which target is the best for inhibiting Rac1 remains to be determined, which may provide a better treatment for breast cancer.

## Data Availability

No datasets were generated or analysed during the current study.
